# Associations between the *CADM2* gene, substance use, risky sexual behavior, and self-control: A phenome-wide association study

**DOI:** 10.1111/adb.13015

**Published:** 2021-02-18

**Authors:** Rachel M. Arends, Joëlle A. Pasman, Karin J.H. Verweij, Eske M. Derks, Scott D. Gordon, Ian Hickie, Nathaniel S. Thomas, Fazil Aliev, Brendan P. Zietsch, Matthijs D. van der Zee, Brittany L. Mitchell, Nicholas G. Martin, Danielle M. Dick, Nathan A. Gillespie, Eco J.C. de Geus, Dorret I. Boomsma, Arnt F.A. Schellekens, Jacqueline M. Vink

**Affiliations:** 1 Department of Psychiatry, Radboud University Medical Center, The Netherlands; 2 Donders Center for Medical Neuroscience, Donders Institute for Brain, Cognition and Behavior, The Netherlands; 3 Tactus Addiction Care, The Netherlands; 4 Behavioural Science Institute, Radboud University, The Netherlands; 5 Faculty of Medicine, Amsterdam Medical Centre and University of Amsterdam, The Netherlands; 6 Genetic Epidemiology, Statistical Genetics and Translational Neurogenomics Laboratories, QIMR Berghofer Medical Research Institute, Australia; 7 Brain and Mind Centre, University of Sydney, Australia; 8 Department of Psychology, Virginia Commonwealth University, Richmond, VA, USA; 9 Faculty of Business, Karbük University, Turkey; 10 Department of African American Studies, Virginia Commonwealth University, Richmond, VA, USA; 11 School of Medicine and School of Psychology, University of Queensland, Australia; 12 Department of Biological Psychology, Vrije Universiteit Amsterdam, The Netherlands; 13 Netherlands Twin Register, The Netherlands; 14 Department of Genetics and Computational Biology, QIMR Berghofer Medical Research Institute, Australia; 15 School of Biomedical Sciences and Institute of Health and Biomedical Innovation, Queensland University of Technology, Australia; 16 Virginia Institute for Psychiatric and Behavior Genetics, Department of Psychiatry, Virginia Commonwealth University, Richmond, VA, USA; 17 Nijmegen Institute for Scientist-Practitioners in Addiction, The Netherlands

**Keywords:** CADM2, multi-cohort, phenome-wide, risky behavior, self-control, substance use

## Abstract

Risky behaviors, such as substance use and unprotected sex, are associated with various physical and mental health problems. Recent genome-wide association studies indicated that variation in the cell adhesion molecule 2 (*CADM2*) gene plays a role in risky behaviors and self-control. In this phenome-wide scan for risky behavior, it was tested if underlying common vulnerability could be (partly) explained by pleiotropic effects of this gene and how large the effects were. Single nucleotide polymorphism (SNP)-level and gene-level association tests within four samples (25 and Up, Spit for Science, Netherlands Twin Register, and UK Biobank and meta-analyses over all samples (combined sample of 362,018 participants) were conducted to test associations between *CADM2*, substance- and sex-related risk behaviors, and various measures related to self-control. We found significant associations between the *CADM2* gene, various risky behaviors, and different measures of self-control. The largest effect sizes were found for cannabis use, sensation seeking, and disinhibition. Effect sizes ranged from 0.01% to 0.26% for single top SNPs and from 0.07% to 3.02% for independent top SNPs together, with sufficient power observed only in the larger samples and meta-analyses. In the largest cohort, we found indications that risk-taking proneness mediated the association between *CADM2* and latent factors for lifetime smoking and regular alcohol use. This study extends earlier findings that *CADM2* plays a role in risky behaviors and self-control. It also provides insight into gene-level effect sizes and demonstrates the feasibility of testing mediation. These findings present a good starting point for investigating biological etiological pathways underlying risky behaviors.

## 1 | INTRODUCTION

Risky behaviors, such as substance use (e.g., nicotine, alcohol, and cannabis) and unprotected sexual contact, are important factors contributing to physical and mental health problems.^2^ As a result, these risk factors for morbidity and mortality^3^ are included in the global Sustainable Development Goals, set up and agreed on by all member states of the United Nations in 2015 to ensure more healthy lives and promote quality of life worldwide.^4^ For instance, substance use contributes to approximately 12% of deaths worldwide,^5^ due to factors such as an increased risk of respiratory and vascular diseases, various forms of cancer, stroke, suicide, or overdose.^6^ Approximately 4% of the global burden of disease, as measured in disability-adjusted life years (DALYs),^7^ is attributable to alcohol and tobacco use and 0.8% to illicit drugs.^5^ Furthermore, risky sexual behavior (e.g., unprotected sexual intercourse with multiple partners) contributes another 6.3% of the total global burden of disease, as it is associated with the risk of sexually transmitted infections (STIs), human immunodeficiency virus (HIV), or cervical cancer.^6,8^

Various studies indicate that risky behavior has a substantial genetic component. For instance, a substantial part of the variation in the initiation of substance use can be explained by genetic factors: alcohol (37%),^9^ nicotine (44%),^10^ and cannabis (40%–48%).^11^ Even higher heritability estimates are shown for substance use disorders, for example, alcohol: 45%–73%,^9,12^ nicotine: 44%–75%,^9,10,12^ and cannabis: 37%–59%.^11,12^ Furthermore, the heritability of risky sexual behavior was estimated by previous research to be around 33%.^13^ It is assumed that different risky behaviors might merely reflect different phenotypic manifestations of (partly) shared underlying genetic vulnerabilities.^14,15^ However, it is largely unknown which genetic and biological mechanisms underpin the heritability of risky behaviors.^16^

Recent large genome-wide association studies (GWASs) have independently implicated a gene located on chromosome 3 encoding cell adhesion molecule 2 (*CADM2*) in various risky behaviors including alcohol (ab)use,^17^ lifetime cannabis use,^1^ number of sexual partners,^17^ and age at first sexual intercourse.^18^ Proteins encoded by *CADM2* are involved in glutamate signaling, GABA transport, and neuron cell–cell adhesion, especially in the prefrontal and anterior cingulate cortices.^19^ These brain regions are well known for their role in cognitive control and motivational salience, which are in turn involved in impulse regulation and self-control.^20,21^

Low self-control, as indexed by high impulsivity, sensation seeking, and disinhibition, has been associated with engaging in risky behavior, including unprotected sexual intercourse^13^ and substance use (initiation) or abuse.^22,23^ A review by Bezdjian et al. showed heritability for different indices of self-control of around 50% across 41 studies including around 27,000 infants, children, adolescents, and adults.^24^ These findings suggest that genetic factors, at least in part, modulate various aspects of self-control. Specifically, *CADM2* has been associated with sensation seeking,^23^ hyperactivity, and impulsivity.^25^ This suggests potential shared heritability between reduced self-control and risky behavior, most likely due to overlapping underlying biological processes.^13,22,23^ As such, reduced self-control might act as intermediate phenotype, linking *CADM2* and various risky behaviors.

Candidate-gene studies have traditionally selected plausible candidate-genes based on a theory on the underlying biological mechanisms, for example, relating the dopamine cascade to ADHD^26^ or substance use.^27^ This approach is limited by current knowledge of the biology of investigated behaviors.^27^ In addition, candidate-gene studies are often restricted by a lack of available data resulting in underpowered or small-scale designs^28^ and examination of only a few (or a single) phenotype(s).^29^ Consequently, these limitations have rendered the candidate-approach largely unsuccessful.^30,31^

We propose to apply GWAS techniques on a single gene, whose candidate-gene status is anchored in a body of (hypothesisfree) GWASs. In this first phenome-wide association study (PHeWAS)^32^ for *CADM2* and risky behavior, the multiple testing burden is much lower than in GWASs, which should increase power. This study aims to establish if power increases substantially enough to detect associations in smaller samples, thereby also providing insight into gene-level effect sizes. By looking at several risky behavior phenotypes concurrently, we furthermore investigate the link between genetic variation in *CADM2* and substance- and sex-related risk behaviors more comprehensively than single phenotype studies. Doing so, we aim to examine if the involvement of *CADM2* in various risky behaviors and self-control related constructs (i.e., pleiotropy, when a single gene influences the expression of multiple phenotypic traits) can explain the potential genetic overlap between various aspects of reduced self-control and multiple risky behaviors. By combining data from four different cohorts and analyzing a range of risky behaviors and indices of self-control, we aim to increase reliability and robustness of findings.^29^ Finally, we explore if reduced self-control might mediate the relationship between *CADM2* and various risky behaviors.

In data across four European ancestry population-based samples from different countries, we tested here whether single nucleotide polymorphisms (SNPs) in *CADM2* are associated with risk behavior, including (1) substance use and abuse (alcohol, tobacco, cannabis, and other drugs), (2) sexual risk behavior (number of sex partners, sexual risk-taking, and age at first sexual intercourse), and (3) indices of reduced self-control (disinhibition, sensation seeking, risk-taking proneness, and ADHD symptoms). We conduct factor analyses to explore common underlying vulnerability factors. Furthermore, we explore whether relationships between *CADM2* and risk behaviors are mediated by a self-control trait.

## 2 | MATERIALS AND METHODS

### 2.1 | Subjects and procedures

Data from 443,693 participants from four different data sources were used, including the Queensland Twin Registry’s “25 and Up” (25Up: *N* = 2,133) study in Australia,^33^ “Spit for Science” (S4S: *N* = 2,994) study in the USA,^34^ the “Netherlands Twin Register” (NTR: *N* = 12,120) repository in The Netherlands,^35^ and the “UK Biobank” (UKB: *N* = 426,446) in the United Kingdom.^36^ Although 25UP and S4S are considerably smaller than the others, they have not been included in previous risk behavior GWAS and have data on phenotypes that were not available in NTR and UKB, making them valuable additions. All studies were performed in accordance with the Declaration of Helsinki and were approved by local ethical committees. Study details are described in articles referenced in the Supplementary Methods section.

### 2.2 | Measures

#### 2.2.1 | Genotyping and quality control

We used available genotyped or imputed SNP information in and around *CADM2* (chr 3 (3p12.1), bp 83,951,945–86,126,470, GRCh37/hg19). Per sample genotyping, imputation and quality control (QC) procedures can be found in Table S1. Variants with a minor allele frequency (MAF) below 1%, a genotype missingness rate above 5%, or deviations from Hardy–Weinberg Equilibrium (HWE) of *p* < 1e-10 were excluded from further analysis. SNPs were aligned with the 1,000 Genomes reference panel (phase 3),^37^ removing ambiguous SNPs and SNPs that had a MAF that diverged more than 0.15 from that in the reference panel. Following these procedures, *n*_*25Up*_ = 297, *n*_*S4S*_ = 2,972, *n*_*NTR*_ = 6,166, and *n*_*UKB*_ = 4,638 SNPs were available and retained for analysis. Genetic data and data on at least one phenotype were available for *N*_25Up_ = 2,133, *N*_S4S_ = 2,994, *N*_NTR_ = 12,120, and *N*_UKB_ = 426,446 individuals (total *N* = 443,693). The per-phenotype sample size range was *N*_25Up_ = 419–2,071, *N*_S4S_ = 503–2,384, *N*_NTR_ = 581–9,432, and *N*_UKB_ = 23,423–362,018 individuals.

#### 2.2.2 | Outcome measures

In this study, we adopted a PHeWAS approach, meaning that we tested the association between *CADM2* and all risk behavior and self-control measures that were available in the datasets. In order to provide an overview of all measures, we grouped them into six categories: lifetime experience with substance use (regarding tobacco, cannabis, and other substances), age at initiation of substance use (regarding alcohol, tobacco, cannabis, and other substances), average substance use level (regarding alcohol and tobacco), regular substance (ab)use (including regular alcohol, tobacco, and cannabis use and any behavioral/substance addiction), sexual risk behavior (including the number of sexual partners, sexual risk-taking, and age at first sexual intercourse), and self-control (including disinhibition, sensation seeking, risk-taking proneness, and symptoms of ADHD). Variables with a total *N* of < 1,000 were excluded as they could not be analyzed due to a lack of statistical power. Preprocessing of the data included combining measures (e.g., across different waves), removing outliers, and excluding inconsistent or invalid response patterns. An overview of all 23 outcome measures included can be found in Table 1. More detailed information about the (cleaning and combining of the) measures is given in Table S2.

### 2.3 | Data analysis

Primary analyses were performed separately within each cohort and combined in meta-analyses. Identical analysis procedures were used in all individual datasets. Phenotype data cleaning, preparation, and descriptive analyses were conducted using the Statistical Package for the Social Sciences (SPSS; version 25).^38^

To test whether *CADM2* SNPs were associated with separate risk behavior outcomes, association analyses were firstly conducted in PLINK (version 1.9).^39^ For dichotomous phenotypes, logistic regression was used; for continuous variables, we used linear regression. Covariates included sex, age, and highest level of education, as we aimed to capture the influence of *CADM2* on risk behavior and self-control that was independent of these factors (e.g., education has shown to be associated both with *CADM2* and risk behavior).^40^ Furthermore, principal components (PCs) for ancestry were included. PCs are used to control for possible stratification effects that arise when a genetic factor and a trait show a spurious correlation due to systematic differences in allele frequencies between groups of different genetic ancestry. We used the PCs as calculated by the institute we received the data from, following their recommendations on how many PCs were appropriate to control for ancestry stratification effects within their specific sample. Because S4S participants were recruited at university, parental rather than own education level was included as a covariate in this sample. In 25Up, S4S, and NTR we used 10 PCs to control for population stratification, while in UKB we included 40 PCs. We controlled for clustering due to genetic relatedness in the twin datasets (25Up and NTR) by using the family option in PLINK and excluded individuals that showed high genetic relatedness in the other datasets (see Table S1).

Second, to assess the overall effect of the variants at the gene level, the association results were analyzed using Multi-marker Analysis of GenoMic Annotation gene-based tests (MAGMA, version 2).^41^ Because not all phenotypes were present in all cohorts, we conducted these analyses separately per cohort. SNPs were mapped to *CADM2* using 1000Genomes phase 3 data. We used the *snp-wise = top* procedure, which is more sensitive when only a small proportion of SNPs in the gene shows an association. To control for the number of phenotypes tested, we computed the Benjamini–Hochberg False Discovery Rate (FDR)^42^ *p*-values within each variable category, using R (version 3.6.2).^43^ When reporting the results, we present uncorrected *p*-values with an asterisk indicating if the FDR-corrected *p*-value was below *p* = .05.

Thirdly, we conducted two meta-analyses for those phenotypes that were present in multiple datasets in order to maximize power to detect associations. The first meta-analysis was performed on the results from the per-cohort gene-based tests using the meta-analysis procedure in MAGMA. This method aggregates the Z-values for the gene-based associations within the individual cohorts while taking sample size into account, in a procedure similar to “normal” meta-analysis. The results give an indication of the strength of the association with *CADM2* across cohorts. The second meta-analysis was used to get per-SNP effects that can be used to estimate the variance in the phenotype explained by SNPs in the gene (*R*^2^). To conduct these meta-analyses, odds ratios for binary outcome variables were converted to betas with corresponding standard errors in the input files and all continuous variables were standardized. The meta-analysis was conducted in METAL^44^ based on standard errors and effect estimates (rather than on sample size) so that β and se(β) could be obtained.

Using the results from the SNP-based meta-analysis, we computed *R*^2^ (the procedure is described in Supplementary Methods II). To give an indication of how the resulting effect size estimates impacted power, we conducted post-hoc power analyses for the meta-analysis. The analysis was conducted based on the observed effect sizes as a function of the minimum and maximum sample size. We used the compromise power analysis option from the G*power package for the F test family with a single predictor.^45^

#### 2.3.1 | Mediation analysis with latent factors

A secondary aim of this study was to test whether the association between *CADM2* and risky behavior would be mediated by one or more indices of self-control. Assuming that latent factors would be stronger measures of underlying risky behavior propensity than the separate phenotypes (and to limit the number of analyses), we used factor scores in the mediation analyses. Assuming that *CADM2* is associated with risky behavior and reduced self-control in general rather than specific behaviors or constructs per se, such latent factors might show stronger relationships with *CADM2*. We used a data-driven approach without a priori specifying the nature of the factors or the number of factors to extract. We expect clustering due to the overlap in the measures, but the actual clustering could differ per sample. We used PC analysis with principal axis factoring (PAF/PFA) including oblique (oblimin) rotation; missing values were replaced with the mean.^46^ The analyses were conducted separately for each cohort and factors with an Eigenvalue >1 that explained >10% of the variance were extracted from the dataset (see Table S3). Subsequently, individual factor scores were computed using regression.

**FIGURE 1:**
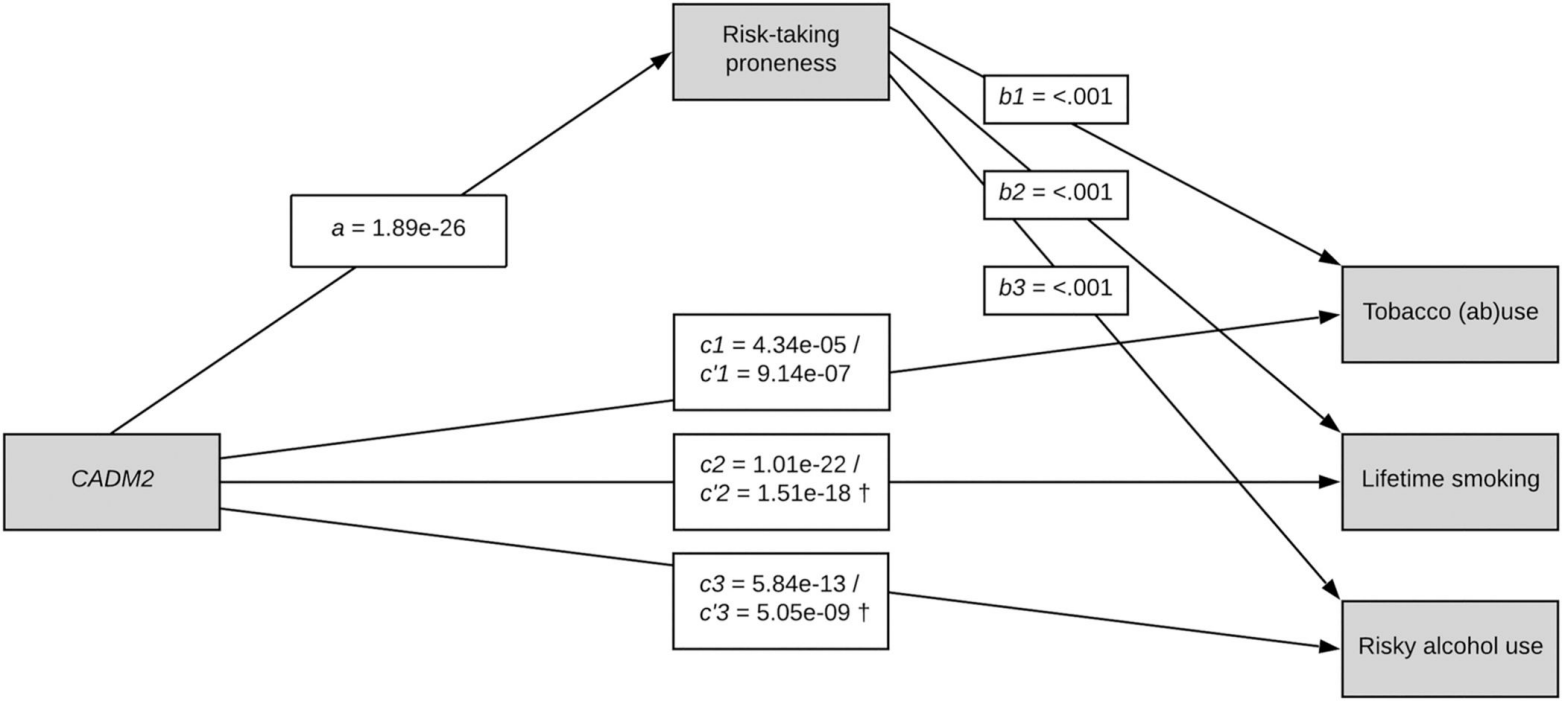
Significance of associations between *CADM2* and risk behavior factors, with and without a mediating effect of risk-taking proneness. Path *a*: the effect of the predictor (*CADM2*) on the mediator (risk-taking proneness); path *b*: the effect of the mediator on the outcome factors (tobacco (ab)use, lifetime smoking, and risky alcohol use); path *c*: the effect of the predictor on the outcome variables; path *c*’: the effect of the predictor on the outcome variables controlling for the mediator. † *C*′ paths with attenuated *p*-values, indicating a partial mediation effect

To test if a self-control trait can explain the association between *CADM2* and risky behavior, we tested mediation following Baron and Kenny’s procedure (see Figure 1, including *p*-values rather than regression weights as MAGMA does not provide such estimates).^47^ We first tested the relationship between *CADM2* and the risk behavior factor (path *c*) in MAGMA, and if that was significant, we tested the association between the self-control trait (mediator) and the risk behavior factor in SPSS (path *b*). If path *b* and path *c* were significant, and there was an association between a self-control trait and *CADM2* in the gene-based test (path *a*), we tested in a final step the relationship between *CADM2* and the risk behavior factor outcome, while controlling for the self-control mediator, in MAGMA (path *c*’). When in path *c*’ the relationship between the risk behavior and *CADM2* was attenuated while controlling for self-control, mediation was assumed.^48^ In all paths, we controlled for the effects of age, sex, and education, and in the analyses involving genetic data, we controlled for the PCs.

As an addition to see if common propensity would indeed show a stronger association with *CADM2*, we also meta-analyzed factors that were made up of similar indicators in different cohorts. We used similar procedures for these analyses as for the separate phenotypes in MAGMA.

## 3 | RESULTS

### 3.1 | Demographics and descriptives

The sample size of people included in at least one analysis consisted of 443,693 individuals (maximum sample size per analysis *N* = 362,018). Slightly more than half of the participants (54%) were female (25Up: 61%, S4S: 58%, NTR: 62%, UKB: 54%), and age ranged from 18 to 94 with a weighted mean age of 38 years (25Up: *M* = 30.1, *SD* = 4.3; S4S: *M* = 20.7, *SD* = 1.5; NTR: *M* = 44.8, *SD* = 16.9; UKB: *M* = 54.7, *SD* = 8.0). Furthermore, most participants had a moderate (49%) or high (33%) level of education (largest group 25Up: 41.7% moderately high, S4S: 77.5% high, NTR: 45.7% high, UKB: 32.4% high education).

Cohort descriptions are provided in Table 1, including a description of the mean (continuous variables) and prevalence rates (dichotomous variables) for all outcome measures. Due to different operationalizations and sample compositions in the four cohorts, most descriptives cannot be directly compared. In the association analyses, we controlled for age, sex, and education level, and we conducted meta-analysis either on per-sample Z-scores for the association (in MAGMA) or on standardized regression weights (in METAL) to control for sample differences.

### 3.2 | Associations for *CADM2* with risk behavior and self-control

The associations between *CADM2* and risk behavior and indices of self-control are shown in Table 2. Associations that were significant after FDR-correction for multiple testing (at *p* < .05) are indicated with an asterisk. Both lifetime tobacco use and lifetime cannabis use were associated with *CADM2* in the meta-analyses. In the individual samples, these associations were significant in NTR and UKB but not in 25Up and S4S. No significant associations were found for lifetime use of other substances (i.e., recreational drugs), although it must be noted that this variable was not present in the largest sample (UKB). None of the age at initiation of substance use variables were associated with *CADM2*. The smallest *p*-value was.049 in the NTR sample for age at alcohol initiation. After correction for multiple testing, this finding was no longer significant. The meta-analyses revealed associations between both average alcohol consumption and average number of cigarettes per day and *CADM2* that seem to be largely driven by significant associations in the UKB sample. Regular alcohol use, problematic alcohol use, regular tobacco use, and nicotine dependence were all associated with *CADM2* in the meta-analyses. In the individual study analyses, only regular alcohol use was after correction significantly associated with *CADM2* in a sample (S4S) other than the UKB. The number of sexual partners was associated with *CADM2* in 25Up, UKB and the meta-analysis, and age at first sexual intercourse in UKB and the meta-analysis but not in the individual 25Up, S4S, or NTR samples.

As for the analyses of indices of self-control, a significant association between *CADM2* and disinhibition (significant in the NTR and meta-analysis), sensation seeking (in NTR), and risk-taking personality (in UKB) was observed. As the constructs of sensation seeking and risk-taking personality were only measured in one study, no meta-analyses could be performed.

SNP-based meta-analyses were conducted in order to get per-SNP estimates that could be used to compute explained variances. Results show little overlap between the top-SNPs for different phenotypes (see Table S4). Only 31 SNPs showed a significant association with multiple independent phenotypes.

#### 3.2.1 | Effect sizes of the associations and power analyses

The variance explained by all independently associated SNPs in *CADM2* taken together ranged from 0.07% for regular alcohol use to 3.02% for regular cannabis use (*M* = 1.05%, *SD* = 1.09%, *Mdn* = 0.45%). The sample sizes included in the analyses ranged from 2,094 to 362,018 individuals (see Table 2). It does not seem to be the case that phenotypes from a particular sample or specific category have higher *R*^2^ than the others. Also, there does not seem to be an effect of the number of SNPs in the analysis on the size of *R*^2^ (*r* = −0.27, *p* > 0.05).

As most effect sizes were below 1%, we set the power analysis parameters at *R*^2^ = 0.001% to 1% as a range for the effect size and 2,000–400,000 as a range for the sample size. For an effect size of 0.001% even a sample size of 400,000 results in a power level of only 50%, whereas for an effect size of 1% a sample size of 8,000 suffices to achieve 80% power. In our study, the average observed effect size of the top SNP was *R*^2^ = 0.11%, resulting in sufficient (>80%) power levels at sample sizes of at least *N* = 7,100. A visualization of power as a function of effect size and the SNP sample size are provided in Figure S1A,B.

#### 3.2.2 | Mediation analysis with latent factors

Factor analysis of the 14–20 outcomes per sample overall identified five factors with Eigenvalues above 1 and explained variance >10%, of which two appeared to be made up by similar variables in multiple cohorts (see Table S3). The latent factor lifetime substance use was present in 25Up and S4S and was not significantly associated with *CADM2*. A tobacco (ab)use factor could be discerned in all datasets but was only significantly associated with *CADM2* in UKB with *p* = 8.45e-06. In UKB there were two other factors, one for lifetime smoking and one for regular alcohol use, which were both associated with *CADM2* (*p* = 1.01e-22 and *p* = 5.84e-13, respectively). Finally, in NTR there was a self-control factor that was associated with *CADM2* (*p* = 2.28e-08).

Thus, there were three risk behavior factors that could be used for the mediation analyses, all extracted from the UKB. There was only one measure of self-control included in the UKB, namely, risk-taking proneness (yes/no). Results of the analysis using this measure as a mediator between *CADM2* and the three risk-taking behavior factors are presented in Figure 1 (with *p*-values rather than regression weights as MAGMA does not provide such estimates). Path a for the association between *CADM2* and risk-taking proneness controlling for sex, age, and PCs was tested earlier and found to be significant (see Table 2). Paths *c1–c3* for the associations between *CADM2* and the outcomes (risk behavior factors) were reported in Table 3. Paths *b1–b3* between risk-taking proneness and the risk behavior factors were all significant (tobacco [ab]use factor OR = 1.27, *p* < .001; lifetime smoking factor, OR = 1.27, *p* < .001; and alcohol abuse factor OR = 1.21, *p* < .001). In step c′, the associations between *CADM2* and lifetime smoking and risky alcohol use factors were attenuated when including the mediator (*p* = 1.01e-22 to 1.51e-18 and 5.84e-13 to 5.05e-09, respectively), suggesting partial mediation by risk-taking proneness. The association between tobacco (ab)use and *CADM2* was enhanced (*p* = 4.34e-05 to 9.14e-07) when controlling for risk-taking proneness, which suggests that there was no mediation effect.

## 4 | DISCUSSION

In this multi-cohort study, it was shown that *CADM2* is associated with multiple substance use and abuse traits, sex-related risky behavior, and different indices of self-control. Meta-analyses showed significant associations between *CADM2* and lifetime experience with tobacco and cannabis use, average alcohol and cigarette consumption, regular/problematic alcohol and tobacco use, number of sexual partners, age at first sexual intercourse, and disinhibition. Furthermore, in the per-sample analyses there were significant associations with sensation seeking, behavioral or substance addiction, and risk-taking proneness. The variance explained by a single *CADM2* SNP ranged from 0.01% (for average alcohol consumption, cigarettes per day, nicotine dependence, and the number of sexual partners) to 0.26% (sensation seeking). Independent top SNPs together explained between 0.07% (regular alcohol use) and 3.02% (regular cannabis use) of the variance. Finally, the self-control trait “risk-taking proneness” was found to be a significant partial mediator of the associations between *CADM2* and latent factors for lifetime smoking and regular alcohol use.

The results of this study are in line with results from recent GWAS, indicating associations of *CADM2* with substance use and abuse (including alcohol consumption, lifetime cannabis use, and general drug experimentation),^1,17,23,49^ sexual risk behavior (such as age at first sexual intercourse and number of sexual partners),^17,28^ and different aspects of self-control (sensation seeking, hyperactivity, and risk-taking propensity).^1,18,23,25^ Our study finds support for these findings in a large, hypothesis-driven, multi-cohort and phenome-wide study for risk behavior, indicating that the role of *CADM2* in risky behaviors and reduced self-control is robust. This is also in line with some earlier reported genetic correlations for various forms of risky behaviors,^40^ suggesting overlapping genes directly or indirectly influence these behaviors. The observed mediation effect of risk-taking proneness is in line with previous suggestions that the association between substance use and *CADM2* might be (partially) mediated by reduced self-control.^49^ Our results suggest that variability in *CADM2* may give rise to various aspects of reduced self-control underlying multiple expressions of risky behavior. This corresponds with proposed shared genetic and neurobiological mechanisms underlying various risky behaviors.^14,15^

*CADM2* is mainly expressed in the brain (predominantly prefrontal and anterior cingulate cortices [PFC and ACC]), the central nervous system, and its peripheral nerve fibers.^23,50^ The PFC and ACC are generally involved in cognitive functions concerned with motivation and controlling behavior.^51^ The ACC has been associated with error detection and response inhibition, whereas several regions within the PFC are involved in reward learning and decision-making processes, which can all be linked to self-control and risky behavior.^52–54^ By affecting brain functions in these regions, variation in *CADM2* may result in different manifestations of reduced self-control and risky behavior. Future research could further delineate which neurobiological mechanisms are involved in the link between *CADM2*, reduced self-control, and risky behaviors.

Looking at the individual SNPs (see Table S4), we observe that most top SNPs cluster in the region roughly around 85,500,000 (see Figure S2). This is a region containing large numbers of expression quantitative trait loci (eQTLs; panel C). eQTLs are places in the genome that influence to what extent a gene comes to expression, that is, how much is transcribed to messenger RNA. Only a few SNPs are among the top 10 independent SNPs for more than one phenotype. This suggests that the effects of *CADM2* were not driven by one strong causal SNP. Six SNPs were associated with three different (but overlapping) primary phenotypes (sensation seeking, any behavioral/substance addiction, and risk-taking proneness). Another SNP that was a top SNP more than twice was rs1271459, associated with ever tobacco use, regular tobacco use, and age at first sexual intercourse. SNPs associated with multiple distinct phenotypes might be more central to the functioning of the gene. As an illustration, we looked up this rs1271459. No information was available for this SNP itself, but its proxy rs9820373 is a significant eQTL for *CADM2* expression in the subcutaneous adipose tissue (*p*_*fdr*_ = 5.4E-4).^55^ This is interesting as *CADM2* has been associated with BMI,^56^ potentially through impulsive over-eating.

### 4.1 | Strengths and limitations

This study has to be viewed in light of its strengths and limitations. Data from separate cohorts with different characteristics were used, which results in a large sample size and high generalizability. It also induces measure heterogeneity, which on the one hand may have limited the power to detect effects in the meta-analyses and on the other hand further substantiates the robustness of findings. This study included a range of risky behavior and self-control phenotypes, potentially expanding the findings. Furthermore, previous research also indicates that *CADM2* may play a role in phenotypically heterogeneous risk-taking behaviors and personality.^1,23^ Future studies might further explore the role of *CADM2* in other potentially related phenotypes, such as (a lack of) physical activity, eating patterns or overweight, gambling, and reckless driving^2^ and should investigate if these results generalize to populations with different age ranges or different genetic ancestry.

In this study, we observed explained variances between 0.01% and 3.02%. The 25UP and S4S samples were too small to detect significant effects in the individual samples. Virtually all phenotypes reached significance only after adding data from the larger samples (NTR and UKB). The comparison of four cohorts with different sample sizes has shown that in general samples of over 7,000 individuals are needed to find significant effects with these effect sizes (see Figure S1).^45,49^ This means that for the phenotypes that were available in UK Biobank, the addition of the other samples has not led to a substantial increase in information over and above what we already learned from previous studies. This is the first study to our knowledge, using this method to give a concrete indication of what sample sizes are needed to detect the effect of a single gene. We may conclude that we must be cautious to draw conclusions from individual small samples, but that these smaller samples can be combined in meta-analyses, especially for (possibly more detailed) phenotypes that are not available in large-scale data sets.

This is the first study aiming to shed light on effect sizes that can be expected on the level of genes. Although small, these effects are substantially larger than those of single variants, as have traditionally been investigated in candidate-gene research. Also, given that behavior arises as a result of a complex interplay between environment and a large number of genes with small effects, the effect sizes of *CADM2* that we find could actually be considered substantial. Looking at the level of genes rather than SNPs is biologically more meaningful and could provide clues on underlying biological mechanisms, which in turn will contribute to a better understanding of transgenerational transmission of risky behaviors and provide clues for designing treatment and prevention programs.

This study shows the feasibility and added value of novel variations of the more common analyses in the field of behavior genetics, including genetic association analyses on factor analyzed traits and mediation analyses. New questions might be answered using such techniques, providing more insight into underlying common vulnerability patterns and etiological mechanisms. However, there were some limitations to the mediation analyses, including the lack of control for family relatedness and covariates in the Principal Components Analyses and the impossibility of calculating regression weights for the associations with *CADM2*. Also, we used Baron and Kenny’s procedure to test for mediation only for outcomes that showed a significant relationship with *CADM2*.^47^ Technically, mediation could arise in the absence of such a relationship. Bootstrapping is a more recently developed non-parametric method that can increase power to detect mediation. However, this approach has not yet been implemented in the area of genetic association analysis. Future research might develop techniques to tackle these limitations. In conclusion, the mediation results in this study suggest that mediation testing may be feasible, but improved statistical tools applicable to behavioral genetics need to be developed.

Next to the genetic etiology of risk behaviors, we recognize the generally known influence of environmental factors.^13^ For example cultural, parenting or peer norms can influence substance- and sex-related risky behaviors. What remains largely unknown is to what extent the impact of genetic and environmental risks is additive or interactive. The variants in *CADM2* identified here lend themselves well to future gene–environment interaction testing, provided a multi-cohort study and a combined SNP measure are used to ensure sufficient power.

## 5 | CONCLUSIONS

This comprehensive multi-cohort study has shown the feasibility of a PHeWAS for risky behavior to confirm previous findings on associations between *CADM2* and manifestations of risky behavior and reduced self-control from GWASs on individual phenotypes. It was shown that single SNPs in *CADM2* could explain 0.01% to 0.26% of the variance and a combination of independent top SNPs together 0.07% to 3.02%. This study provides more insight into the relatively small effect sizes that can be expected from association studies. Furthermore, results revealed that a self-control trait might partially mediate the associations between *CADM2* and substance-related risky behavior (lifetime smoking and regular alcohol use). Future studies should further explore the biological underpinnings of the observed relationships between *CADM2*, reduced self-control, and various risky behaviors.

## Supplementary Material

Supplement 1

## Figures and Tables

**TABLE 1:** Descriptive statistics of participant characteristics and study variables for the four cohort studies

	25Up (*N* = 2,133)			S4S (*N* = 2,994)			NTR (*N* = 12,120)			UKB (*N* = 426,446)		
Variable	*N*	Range/*n*	*M* (*SD*) / %	*N*	Range/*n*	*M* (SD) / %	*N*	Range/*n*	*M* (*SD*) / %	*N*	Range/*n*	*M* (*SD*) / %
**Demographics**												
Age	2,131	21.8–44.2	30.1 (4.3)	2,452	18.1–32.1	20.7 (1.5)	12,013	18.0–94.0	44.8 (16.9)	426,446	36.0–72.0	54.7 (8.0)
% Male	2,133	832	39.0%	2,994	1,255	41.9%	12,120	4,564	37.7%	426,446	196,462	46.1%
% No/primary education ^a^	2,132	250	11.7%	2,963	5	0.2%	9,471	295	3.1%	426,446	75,577	17.7%
% Secondary education		572	26.8%		238	8.0%		1,954	20.6%		114,042	26.7%
% Certificate or diploma		890	41.7%		425	14.3%		2,892	30.5%		98,540	23.1%
% Masters/postgraduate/PhD		420	19.7%		2,295	77.5%		4,330	45.7%		138,287	32.4%
**Lifetime substance use**												
% Ever used tobacco	2,128	1,496	70.3%	2,876	1,949	67.8%	11,870	5,745	48.4%	425,114	259,676	61.1%
% Ever used cannabis	2,118	1,331	62.8%	2,953	1,966	66.6%	10,012	2,412	24.1%	138,524	30,627	22.1%
% Ever used other substance(s) ^b^	2,062	1,104	53.5%	2,968	1,067	36.0%	10,055	1,153	11.5%	NA	NA	NA
**Age at initiation of substance use**												
*M* age alcohol initiation	1,995	11–33	16.1 (1.9)	2,469	11–22	16.5 (1.8)	9,762	11–30	15.8 (2.1)	NA	NA	NA
*M* age tobacco initiation	471	11–34	17.7 (3.5)	1,554	11–21	15.9 (2.0)	6,862	11–30	15.4 (2.3)	32,381	11–69	17.9 (5.7)
*M* age cannabis initiation	1,275	12–36	18.4 (3.5)	1,393	11–19	14.4 (2.0)	9,729	11–40	17.9 (3.3)	NA	NA	NA
*M* age other substance initiation	1,037	11–37	20.2 (3.9)	617	11–24	15.1 (2.1)	11,381	11–40	21.4 (5.2)	NA	NA	NA
**Average substance use**												
*M* alcohol units per month	1,929	1–395	25.2 (37.7)	2,594	0–199	23.1 (30.4)	8,670	0–192	34.9 (27.3)	304,654	0–200	8.8 (7.8)
*M* cigarettes per day	430	0–30	5.3 (7.4)	1,422	1–4	1.3 (0.6)	4,819	0–60	13.8 (9.4)	132,310	1–140	18.3 (10.1)
*M* tobacco using days	430	1–30	14.8 (13.4)	1,817	1–28	6.4 (8.5)	2,016	4–30	23.7 (9.9)	NA	NA	NA
**Regular substance (ab)use**												
% Regular alcohol use	2,062	450	21.8%	2,662	220	8.3%	11,081	2,722	24.6%	138,693	42,564	30.7%
*M* problematic alcohol use (AUDIT)	NA	NA	NA	2,603	0–11	3.5 (2.7)	6,248	0–34	4.1 (3.6)	138,682	0–39	5.0 (4.2)
% Regular tobacco use	1,490	489	32.8%	2,979	427	14.3%	8,051	2,386	29.6%	299,866	133,640	44.6%
*M* nicotine dependence (FTND)	NA	NA	NA	1,553	0–9	1.1 (1.9)	4,475	0–10	2.8 (2.4)	132,541	0–9	1.8 (1.9)
% Regular cannabis use	1,317	303	23.0%	2,953	397	13.4%	1,493	232	15.5%	29,704	10,465	35.2%
% Any behavioral/substance addiction	NA	NA	NA	NA	NA	NA	NA	NA	NA	137,122	8,278	6.0%
**Sexual risk behavior**												
*M* number of sexual partners	1,719	0–300	13.3 (24.4)	1,023	0–25	1.3 (1.8)	NA	NA	NA	351,099	1–300	6.6 (13.0)
*M*/% sexual risk behavior	1,925	0–10	2.1 (2.4)	1,014	375	37.0%	NA	NA	NA	NA	NA	NA
*M* age at first sexual intercourse	NA	NA	NA	919	11–28	16.3 (3.1)	1,071	12–18	17.1 (1.2)	375,311	11–69	19.1 (3.8)
**Self-control**												
*M* disinhibition	NA	NA	NA	2,757	1–18	11.0 (2.1)	9,785	5–25	14.2 (3.7)	NA	NA	NA
*M* sensation seeking	NA	NA	NA	NA	NA	NA	9,229	4–20	11.3 (2.5)	NA	NA	NA
% risk-taking proneness	NA	NA	NA	NA	NA	NA	NA	NA	NA	412,571	111,571	27.0%
*M*/% ADHD	2,051	7–88	39.0 (10.4)	657	106	16.1%	8,046	0–12	2.3 (1.9)	NA	NA	NA

*Note:* The variations in sample sizes are due to question branching and the pooling of data of several measurement waves. *N* = total number of participants with phenotypic and genetic data available per variable and subsample; range = minimum and maximum score/answer; *n* = number of participants scoring positive on this variable within the subsample; *M* = mean within this cohort; *SD* = standard deviation within this cohort; % = percentage of the total number of participants in a subsample.

Abbreviations: 25Up, 25 and Up; ADHD, attention deficit-/hyperactivity disorder; AUDIT, Alcohol Use Disorders Identification Test; FTND, Fagerström Test for Nicotine Dependence; NTR, Netherlands Twin Register; S4S, Spit for Science; UKB, UK Biobank.

a Education levels of participants or their parents in the S4S study.

b Includes the use of other substances than alcohol, tobacco, and cannabis.

**TABLE 2:** Results from the MAGMA gene-based tests per sample, with meta-analysis results for variables that were present in two or more datasets

	25Up		S4S		NTR		UKB		Meta-analysis				
Variable	*p*	*N*	*p*	*N*	*p*	*N*	*p*	*N*	*p*	*N*	% *R*^2^ top	#SNPs	% *R*^2^
**Lifetime substance use**													
Ever used tobacco	0.919	2,071	0.601	2,279	**0.002** *	9,432	**1.12e-20** *	348,237	**2.23e-21** ^*^	362,018	0.18%	63	0.78%
Ever used cannabis	0.424	2,061	0.502	2,374	**0.008** *	8,022	**2.30e-17** *	128,132	**3.51e-18** *	140,588	0.06%	83	2.28%
Ever used other substance(s)	0.858	2,008	0.184	2,380	0.179	8,073	-	-	0.241	12,460			
**Age at initiation of substance use**													
Age alcohol initiation	0.818	1,940	0.887	2,048	**0.049**	7,784	-	-	0.318	11,772			
Age tobacco initiation	0.929	458	0.737	1,343	0.370	5,664	0.444	23,423	0.519	30,888			
Age cannabis initiation	0.587	1,247	0.139	1,130	0.820	1,877	-	-	0.568	4,254			
Age other substance initiation	0.172	1,010	0.922	503	0.443	581	-	-	0.485	2,094			
**Average substance use**													
Average alcohol units	0.678	1,876	0.274	2,169	0.202	7,211	**1.71e-07** *	257,221	**1.35e-07** *	268,477	0.01%	69	0.17%
Average cigarettes per day	**0.021**	419	0.177	1,242	0.429	4,016	**0.002** *	100,604	**0.001** *	106,281	0.01%	53	0.27%
Average tobacco using days	0.178	419	0.632	1,505	0.216	1,633	-	-	0.264	3,557			
**Regular substance (ab)use**													
Regular alcohol use	0.605	2,007	**0.010** *	2,275	0.076	8,927	**4.07e-04** *	128,294	**6.95e-05** *	141,503	0.04%	52	0.07%
Problematic alcohol use (AUDIT)	-	-	0.351	2,151	0.536	5,369	**8.52e-12** *	128,286	**2.50e-11** *	135,806	0.02%	67	0.31%
Regular tobacco use	0.787	1,455	0.829	2,384	**0.021**	6,634	**5.10e-20** *	240,850	**5.84e-20** *	251,323	0.02%	90	1.22%
Nicotine dependence (FTND)	-	-	**0.047**	1,330	0.546	3,831	**7.37e-05** *	100,730	**5.50e-05** *	105,891	0.01%	59	0.31%
Regular cannabis use	0.710	1,288	0.155	2,373	0.999	1,216	**0.033** *	28,800	0.110	33,677	0.04%	50	3.02%
Any behavioral/substance addiction	-	-	-	-	-	-	**0.001** *	126,817	-	-	0.04%	37	0.66%
**Sexual risk behavior**													
Number of sexual partners	**0.023** *	1,677	0.468	997	-	-	**2.31e-06** *	295,706	**1.21e-06** *	298,380	0.01%	54	0.12%
Sexual risk behavior	0.673	1,873	0.529	990	-	-	-	-	0.657	2,863			
Age at first sexual intercourse	-	-	0.173	896	0.416	941	**2.86e-18** *	315,749	**2.08e-18** *	317,586	0.02%	91	0.30%
**Self-control**													
Disinhibition	-	-	0.612	2,316	**4.36e-04** *	8,169	-	-	**0.003** *	10,485	0.16%	45	2.47%
Sensation seeking	-	-	-	-	**8.00e-07** *	7,667	-	-	-	-	0.26%	34	2.66%
Risk-taking proneness	-	-	-	-	-	-	**1.89e-26** *	338,031	-	-	0.03%	68	0.21%
ADHD	0.136	1,995	0.533	647	0.088	6,525	-	-	0.052	9,167			

*Note:* Explained variance is given for the top SNP and the independent SNPs together, for significant associations in the meta-analysis (or cohort analysis for phenotypes present in only one cohort), *p* = MAGMA *p*-value (when bold: *p* < 0.05, when bold and underscored: *p* < 0.01.)

* = *p*-value is also significant when corrected for false discovery rate (FDR); *N* = sample size per variable and subsample; %*R*^2^ top = percentage of variance explained by the top SNP; #SNPs = number of independent SNPs in *CADM2* (LD *R*^2^ = 0.1%) included in the meta-analysis; % *R*^2^ based on those independent SNPs (SNP IDs are shown in Table S4).

Abbreviations: 25Up, 25 and Up; ADHD, attention deficit-/hyperactivity disorder; AUDIT, Alcohol Use Disorders Identification Test; FTND, Fagerström Test for Nicotine Dependence; MAGMA, Multi-marker Analysis of GenoMic Annotation; NTR, Netherlands Twin Register; S4S, Spit for Science; SNP, single nucleotide polymorphism; UKB, UK Biobank.

**TABLE 3:** Results for gene-based analyses between *CADM2* and factors from the Principal Components Analyses

	25Up		S4S		NTR		UKB		Meta-analysis				
Factor	*p*	*N*	*p*	*N*	*p*	*N*	*p*	*N*	*p*	*N*	% *R*^2^ top	#SNPs	% *R*^2^
Lifetime substance use^a^	0.581	2076	0.733	2,389					0.723	4,465	0.09%	12	0.58%
Tobacco (ab)use^b^	0.057	2076	0.085	2,389	0.084	9,471	**4.34e-05**	348,950	**8.45e-06**	362,886	0.13%	44	2.47%
Lifetime smoking^c^							**1.01e-22**	348,950			0.02%	80	0.24%
Risky alcohol use^d^							**5.84e-13**	348,950			0.01%	63	0.10%
Self-control^e^					**2.28e-08**	9,471					0.24%	44	2.76%

*Note:* Factors were extracted if they had an eigenvalue above 1 and explained ≥10% of the variance in the data, resulting in five factors in total, of which two could be detected in multiple cohorts. Below the table, the factor indicators (variables with factor loading >0.4 on the factor) per sample are given. *p* = MAGMA *p*-value (when bold: *p* < 0.05, when bold and underscored: *p* < 0.01.); *N* = sample size per factor and cohort; %*R*^2^ top = percentage of variance explained by the top SNP; #SNPs = number of independent SNPs in *CADM2* (LD *R*^2^ = 0.1%) included in the meta-analysis; % *R*^2^ based on those independent SNPs (SNP IDs are shown in Table S4).

Abbreviations: 25Up, 25 and Up; NTR, Netherlands Twin Register; MAGMA, Multi-marker Analysis of GenoMic Annotation; S4S, Spit for Science; SNP, single nucleotide polymorphism; UKB, UK Biobank.

a Lifetime substance use was defined by ever used cannabis, ever used tobacco and ever used other substances in 25UP and by ever used cannabis and ever used other substances in S4S.

b Tobacco (ab)use was defined by nicotine dependence and average cigarettes per day in 25UP, by regular tobacco use, average tobacco using days and ever used tobacco in S4S, by the same variables in NTR, and by nicotine dependence, average cigarettes per day, and age at smoking initiation in UKB.

c Lifetime smoking was defined by regular tobacco use and ever used tobacco in UKB.

d Risky alcohol use was defined by problematic alcohol use (AUDIT), regular alcohol use, and average alcohol units per month in UKB.

e Self-control was defined by disinhibition, sensation seeking, and ADHD in NTR.
