# Supplementary material for: Associations between the CADM2 gene, substance use, risky sexual behavior, and self-control: A phenome-wide association study
Source: Addict Biol. Author manuscript; Available in PMC 2022 Nov 1. (PMC8596397; doi:10.1111/adb.13015)
Supplement: Supplement 1 [file NIHMS1752320-supplement-Supplement_1.docx]

**SUPPLEMENTARY INFORMATION**

**Supplementary methods I – Study details**

25Up study

This study is an extension of previous Brisbane Longitudinal Twin Studies. The data was collected between 2016 and 2018 from twins and their non-twin siblings. The main aim of these studies was to longitudinally assess genetic, psychological, behavioral, and demographic risk factors for various mental disorders in a large cohort of Australian twins and their non-twin siblings, by using questionnaire, genome-wide genetic and pedigree information. Content and methods of the 25Up study are further described in a recent article.^1^

S4S study

The Spit for Science (S4S) study is a prospective, longitudinal study of the genetic and environmental influences on behavioral and emotional health among undergraduate students at a large urban university in the mid-Atlantic region.^2^ The study examined questions concerning for instance demographic topics, mental health, life experiences, and various (risk) behaviors. In total, there were 7 waves of data collection used in this study. We selected adult Caucasian students that had survey and genetic data available.

NTR study

This sample comprised participants registered at the Netherlands Twin Register,^3^ an ongoing longitudinal study of Dutch twins and their family members. Survey questions encompass topics as physical and mental health, lifestyle, and personality. NTR participants were included in this study for whom genotype data were available and who completed questions related to risk behavior (substance use and sexual risk behavior) and indices of self-control in one or more waves of the longitudinal survey project. We used data from the adult sample (ANTR) wave 1 to 8 (1991-2010) and 10 (2013-2014) to 12 (2013-2016) and the young (YNTR) sample at age 18. DNA collection procedures have been described elsewhere.^3^

UKB study

The UK-Biobank constitutes a large nationwide effort to follow the (psychological) health of half a million UK individuals.^4^ Genetic, behavioral, medical, and imaging data were collected from 2006 onwards, and more data is still being collected. Data from participants was included in this study if genetic information was available and someone filled in at least one questionnaire on one wave (regarding substance use, sexual risk factors or self-control).

**Supplementary methods II – Procedure for estimating effect size**

Using the results from the SNP-based meta-analysis we computed explained variance based on previously reported procedures.^5^ For continuous phenotypes, we used the formula *R^2^*^=^$\frac{2\beta^{2}MAF(1-MAF)}{2\beta^{2}MAF\left( 1-MAF \right)+{(se\left( \beta\right))}^{2}2N MAF(1-MAF)}$ to estimate the explained variance of the top SNP, with *MAF* being the minor allele frequency from the 1000 Genomes reference panel^6^, *β* the effect estimate from the meta-analysis, and *se* the corresponding standard error.^7^ For the binary phenotypes, we first estimated the *t*-value corresponding to the *p-*value using the quantile function of the student *t-*distribution, with the degrees of freedom based on the effective sample size N*=*$4/(\frac{1}{N_{cases}}+\frac{1}{N_{controls}})$, and calculated *R^2^*^=^${(\frac{t}{\sqrt{N+t^{2}}-2})}^{2}$. To check if considering multiple SNPs in the gene would increase explained variance we calculated and summed *R^2^* for independent SNPs (*r^2^*≤0.10%) that showed an association with two-sided *p*<.100. This lenient threshold was used in order to integrate effect sizes that did not reach significance in the smaller cohorts, in an approach equivalent to what is often done for polygenic risk scores.^8^ For the binary phenotypes, the summed *R^2^* was approached using $\frac{2MAF(1-MAF)\beta^{2}}{var(y)}$ with var(y)= $\frac{2MAF(1-MAF)\beta^{2}}{R^{2}}$ from the top SNP.

**Supplementary tables**

**SUPPLEMENTARY TABLE S1** Genotyping and imputation procedures used

|  | **25Up** | **S4S** | **NTR** | **UKB** |
| --- | --- | --- | --- | --- |
| **bp start (GRCh37/hg19)** | 83,901.945‬ | 83,951,945 | 84,068,424 | 83,951,956 |
| **bp stop (GRCh37/hg19)** | 85,568,580 | 86,126,470 | 86,031,960 | 86,126,239 |
| **genotyping platform** | Illumina 610k SNP | Affymetrix BioBank | Affymetrix 6.0^a^  Illumina 660  Illumina 1M  Perlegen-Affymetrix  Typed using GONL sequence data | UK-Biobank Axiom UKBiLEVE |
| **imputation platform** | PLINK  HapMap | SHAPEIT2/ IMPUTE2  1000 genomes | Minimac3/ Eagle  HRC | SHAPEIT3/ IMPUTE 4  HRC/ 1000 genomes / UK10K |
| **sample QC procedures** | Described by Gillespie et al.^9^ | Described by Peterson et al.^10^ | Described by Willemsen et al.^3^ | Described by Bycroft et al.^11^ |
| **relatedness threshold** | NA^b^ | $\hat{\pi}$ = 0.35 | NA^b^ | KING = .0625 |
| **N_ethnic_outliers_** | 74 | 3,307 | 1,591 | 63,144 |
| **N_individuals_ after QC** | 2,133 | 2,994 | 12,120 | 426,446 |
| **N_SNPs_ before QC** | 321 | 3,312 | 7,021 | 7,290 |
| **N_SNPs_ after QC** | 297 | 2,972 | 6,166 | 4,638 |

NA = not applicable; QC = quality control; PMID = PubMed identifier.

^a^ Genotyping platform was included as a covariate in the NTR association analyses.

^b^ In the family-based samples from 25Up and NTR related individuals were not excluded (the analyses controlled for family structure in the data).

**SUPPLEMENTARY TABLE S2** Overview of included variables and used measures in the 25Up, S4S, NTR, and UKB studies. For each construct the composite measure, the number of items it was based on, and the number of times these items were measured. If an item was measured on multiple waves, we checked the consistency of reporting where possible (e.g. age at initiation variables) and took the highest or average of the reported instances (indicated with ‘maximum’ or ‘average’ in the table) depending on the type of measure. Outliers and unreliable response patterns were removed and data were standardized before analysis

| **Variable** | **25Up** | **S4S** | **NTR** | **UKB** |
| --- | --- | --- | --- | --- |
| **DEMOGRAPHICS** |  |  |  |  |
| Age | Maximum score: 1 item  age at last survey completion | Maximum score: 1 item (7 waves)  age at last survey completion | Maximum score: 1 item (12 waves)  age at last survey completion | Raw score: 1 item (1 wave)  year of birth |
| Sex | Biologically determined sex | Biologically determined sex | Biologically determined sex |  |
| Education | Raw score: 1 item  highest (partially) completed  education level | Maximum score: 2 items (1 wave)  education level mother  education level father | Maximum score: 1 item (12 waves)  completed education level | Maximum score: 1 item (6 waves)  educational qualifications |
| **LIFETIME SUBSTANCE USE** | | | | |
| Ever used tobacco | Dichotomous ever/never: 1 item  ever tried tobacco product | Dichotomous ever/never: 4 items (7 waves)  lifetime smoking  smoking frequency  nicotine dependence  age at smoking initiation | Dichotomous ever/never: 1-5 items (12 waves)  lifetime smoking  smoking frequency (also in ex-  smokers)  tobacco products per day (also in  ex-smokers)  nicotine dependence | Dichotomous ever/never: 4 items (3 waves)  smoking status  ever smoking  pack years of smoking (number of  packs p.d.*number of years  smoking)  proportional pack years of  smoking |
| Ever used cannabis | Dichotomous ever/never: 1 item  ever tried cannabis | Dichotomous yes/no: 1 item (7 waves)  past year cannabis use | Dichotomous ever/never: 1-3 items (5 waves)  lifetime use  age at first (regular) use  past year use | Dichotomous ever/never: 2 items (1 waves)  ever taking cannabis  maximum use frequency |
| Ever used other substance(s) | Dichotomous ever/never: 13 items  ever tried a specific drug | Dichotomous ever/never: 3 items (3 waves)  past year stimulant use  past year cocaine use  past year opioid use | Dichotomous ever/never: 1-16 items (6 waves)  lifetime (experimental) use of  specific drug or drug category  regular use  age at first (regular) use | NA |
| **AGE AT INITIATION OF SUBSTANCE USE** | | | | |
| Age alcohol initiation | Raw score: 1 item  age first full-serve alcohol | Average score: 1 item (7 waves)  age first time drunk | Average score: 1 item (7 waves)  age at first alcohol use | NA |
| Age tobacco initiation | Raw score: 1 item  age at daily smoking initiation | Average score: 1 item (6 waves)  age first cigarette | Average score: 1 item (7 waves)  age first cigarette | Average score: 1 item (2 waves)  age at (regular) smoking initiation |
| Age cannabis initiation | Raw score: 1 item  age at first use cannabis | Average score: 1 item (1 wave)  age at first use cannabis | Average score: 1 item (5 waves)  age first use cannabis/ soft drug | NA |
| Age other substance initiation | Raw score: 13 items  age at first use specific drugs | Average score: 3 items (1 wave)  age at first use stimulant  age at first use cocaine  age at first use opioid | Average score: 2-8 items (5 waves)  age first (experimental) use of  specific drug or drug category | NA |
| **AVERAGE SUBSTANCE USE** | | | | |
| Average alcohol units per month | Total score: 2 items  alcohol use frequency  glasses consumed per drinking  occasion | Average score: 1 item (7 waves)  glasses consumed per month | Average score: 1-2 items (10 waves)  glasses of alcohol on weekdays  glasses of alcohol on weekend  days | Average total score: 6 items (3 waves)  weekly red wine  weekly champagne/ white wine  weekly beer/ cider  weekly spirits  weekly fortified wine  weekly other alcohol |
| Average cigarettes per day | Raw score: 1 item  tobacco products used per day | Maximum score: 1 item (7 waves)  cigarettes smoked per day | Maximum score: 1-4 items (11 waves)  cigarettes per day for current  smokers  cigarettes per day at the period of  heaviest smoking for current and  ex-smokers  tobacco products per day | Average score: 2 items (3 waves)  cigarettes per day for current  smokers  cigarettes per day for ex-smokers |
| Average tobacco using days | Total score: 1 item  tobacco using days per year | Average score: 1 item (7 waves)  tobacco use frequency | Average score: 1 item (6 waves)  smoking frequency | NA |
| **REGULAR SUBSTANCE (AB)USE** | | | | |
| Regular alcohol use | Dichotomous yes/no: 1 item  drinking ≥ 4 times per week | Dichotomous ever/never: 1 item (7 waves)  drinking ≥4 times per week | Dichotomous ever/never: 1-2 items (10 waves)  drinking ≥ 4 times per week | Dichotomous yes/no: 1 item (1 wave)  drinking ≥ 4 times per week |
| Problematic alcohol use (AUDIT) | NA | Maximum score: 1 item (7 waves)  total score AUDIT | Total score: 1 item (1 wave)  total score AUDIT | Total score: 1 item (1 wave)  total score AUDIT |
| Regular tobacco use | Dichotomous ever/never: 1 item  daily smoking | Dichotomous ever/never: 1 item (7 waves)  daily smoking  cigarettes per day | Dichotomous ever/never: 1 item (6 waves)  daily smoking  cigarettes per day | Dichotomous ever/never: 2 items (3 waves)  daily smoking  cigarettes per day |
| Nicotine dependence (FTND) | NA | Maximum score: 1 item (7 waves)  total score FTND | Maximum score: 1-2 items (5 waves)  total score FTND in ever smokers  total score FTND in ex-smokers  total score FTND in current  smokers | Maximum sum score: 3 items (3 waves)  total score FTND based on 3 items  (time to first cigarette, difficulty  To refrain, cigarettes per day) |
| Regular cannabis use | Dichotomous ever/never: 1 item  used ≥ weekly | Dichotomous reported at least twice/ less: 1 item (7 waves)  used ≥ 6 times a year | Dichotomous ever/never: 1 item (3 waves)  regular use  age at regular use | Dichotomous yes/no: 1 item (1 wave)  at least monthly cannabis use |
| Any behavioral/substance addiction | NA | NA | NA | Dichotomous yes/no: 1 item (1 wave)  ever addicted to substance or  behavior |
| **SEXUAL RISK BEHAVIOR** | | | | |
| Number of sexual partners | Raw score: 1 item  number of lifetime sexual  partners | Maximum score: 1 item (2 waves)  number of sex partners past 3  months | NA | Average score: 1 item (3 waves)  number of lifetime sexual  partners |
| Sexual risk behavior | Total score: 2 items  no need for affection in sex  prefer several sexual  relationships at once | Dichotomous ever/never: 4 items (2 waves)  unprotected sex while not in  relationship  unprotected sex with multiple  people  sex under the influence of drugs  sex under the influence of alcohol | NA | NA |
| Age at first sexual intercourse | NA | Average score: 1 item (2 waves)  age at first time sex | Average score: 1 item (2 waves)  age at first time sex | Average score: 1 item (3 waves)  age at first time sex |
| **SELF-CONTROL** | | | | |
| Disinhibition | NA | Maximum score: 1 item (1 wave)  impulsivity total score (UPPS-P) | Maximum score: 1 item (6 waves)  disinhibition (subscale from SSS) | NA |
| Sensation seeking | NA | NA | Maximum score: 1 item (6 waves)  sensation seeking total score (SSS) | NA |
| Risk-taking proneness | NA | NA | NA | Maximum score: 1 item (3 waves)  inclined to take risks |
| ADHD | Total score: 1 item  ADHD symptom score (ASRS) | Dichotomous ever/never: 2 items (3 waves)  ever diagnosed with ADHD  taking medication for ADHD | Maximum score: 1 item (3 waves)  ADHD symptom score (ASRS) | NA |

*Note.* ADHD/ADD= attention deficit (hyperactivity) disorder; ASRS= ADHD Self-Report Scale^12^; AUDIT= Alcohol Use Disorders Identification Test^13^; FTND= Fagerström Test for Nicotine Dependence^14^; UPPS-P= Urgency, Premeditation (lack of), Perseverance (lack of), Sensation Seeking, Positive Urgency, Impulsive Behavior Scale,^15^ SSS= Sensation Seeking Scale.^16^

**SUPPLEMENTARY TABLE S3** Results of factor-analyses including explained variances and pattern matrices of **(A)** 25Up, **(B)** S4S, **(C)** NTR and **(D)** UKB study data. Pattern matrices show results for factors with eigenvalues ≥ 1.00 indicating more explained variance than a single observed variable, and variable loadings < .30 are not considered significant ^17^ and therefore not reported.

**A)** 25Up data.

| **Factor** | **Eigenvalue** | **% of Variance** | **Cumulative %** |
| --- | --- | --- | --- |
| 1 | 2.84 | 17.75 | 17.75 |
| 2 | 1.98 | 12.34 | 30.09 |
| 3 | 1.47 | 9.17 | 39.26 |
| 4 | 1.35 | 8.43 | 47.70 |
| 5 | 1.08 | 6.76 | 54.45 |
| 6 | 1.05 | 6.57 | 61.02 |
|  |  |  |  |

| **Variable** | **Factor** | | | | | |  | |
| --- | --- | --- | --- | --- | --- | --- | --- | --- |
|  | **1** | **2** | **3** | **4** | **5** | **6** | | **Factor label** |
| Ever used cannabis | .90 |  |  |  |  |  | | Lifetime substance use |
| Ever used tobacco | .69 |  |  |  |  |  | |  |
| Ever used other substance(s) | .40 |  |  |  |  |  | |  |
| Age alcohol initiation | -.37 |  |  |  |  |  | |  |
| Average cigarettes per day |  | .90 |  |  |  |  | | Tobacco (ab)use |
| Average tobacco using days |  | .84 |  |  |  |  | |  |
| Age cannabis initiation |  |  | .60 |  |  |  | | Age at initiation of substance use |
| Age other substance initiation |  |  | .34 |  |  |  | |  |
| Age tobacco initiation |  |  | .33 |  |  |  | |  |
| Regular alcohol use |  |  |  | .79 |  |  | | Alcohol (ab)use |
| Average alcohol units per month |  |  |  | .77 |  |  | |  |
| Regular tobacco use |  |  |  |  | .76 |  | | NA |
| Regular cannabis use |  |  |  |  | .35 |  | |  |
| ADHD |  |  |  |  | * |  | |  |
| Number of sexual partners |  |  |  |  |  | .55 | | Sexual risk behavior |
| Sexual risk behavior |  |  |  |  |  | .51 | |  |
| * Variable loading < .30 | | | | | |  | |  |

| **Factor** | **Eigenvalue** | **% of Variance** | **Cumulative %** |
| --- | --- | --- | --- |
| 1 | 4.10 | 20.52 | 20.52 |
| 2 | 2.10 | 10.52 | 31.04 |
| 3 | 1.30 | 6.49 | 37.52 |
| 4 | 1.24 | 6.19 | 43.72 |
| 5 | 1.20 | 5.98 | 49.70 |
| 6 | 1.04 | 5.20 | 54.90 |
| 7 | 1.02 | 5.08 | 59.97 |

| **Variable** | **Factor** | | | | | | | |  |
| --- | --- | --- | --- | --- | --- | --- | --- | --- | --- |
|  | **1** | **2** | **3** | **4** | **5** | **6** | **7** | **Factor label** | |
| Ever used cannabis | .73 |  |  |  |  |  |  | Lifetime substance use | |
| Ever used tobacco | .72 |  |  |  |  |  |  |  | |
| Ever use other substance(s) | .45 |  |  |  |  |  |  |  | |
| Disinhibition | * |  |  |  |  |  |  |  | |
| Nicotine dependence (FTND) |  | .78 |  |  |  |  |  | Tobacco (ab)use | |
| Average cigarettes per day |  | .71 |  |  |  |  |  |  | |
| Risk score ADHD |  | * |  |  |  |  |  |  | |
| Average alcohol units per month |  |  | .82 |  |  |  |  | Alcohol (ab)use | |
| Regular alcohol use |  |  | .60 |  |  |  |  |  | |
| Problematic alcohol use (AUDIT) |  |  | .39 |  |  |  |  |  | |
| Number of sexual partners |  |  |  | .67 |  |  |  | Sexual risk behavior | |
| Sexual risk behavior |  |  |  | .45 |  |  |  |  | |
| Age first sexual intercourse |  |  |  | * |  |  |  |  | |
| Age tobacco initiation |  |  |  |  | .59 |  |  | Age at initiation of substance use | |
| Age alcohol initiation |  |  |  |  | .51 |  |  |  | |
| Age cannabis initiation |  |  |  |  | * |  |  |  | |
| Age other substance initiation |  |  |  |  | * |  |  |  | |
| Regular tobacco use |  |  |  |  |  | .80 |  | Tobacco (ab)use | |
| Average tobacco using days |  |  |  |  |  | .73 |  |  | |
| Regular cannabis use |  |  |  |  |  |  | -.64 | - | |
| * * Variable loading < .30 | | | | | | | | |  |

**B)** S4S data.

**C)** NTR data.

| **Factor** | **Eigenvalue** | **% of Variance** | **Cumulative %** |
| --- | --- | --- | --- |
| 1 | 2.72 | 14.33 | 14.33 |
| 2 | 2.06 | 10.87 | 25.20 |
| 3 | 1.65 | 8.66 | 33.85 |
| 4 | 1.42 | 7.47 | 41.32 |
| 5 | 1.22 | 6.40 | 47.72 |
| 6 | 1.12 | 5.87 | 53.59 |
| 7 | 1.02 | 5.36 | 58.95 |

| **Variable** |  | **Factor** | | | | | | |
| --- | --- | --- | --- | --- | --- | --- | --- | --- |
|  | **1** | **2** | **3** | **4** | **5** | **6** | **7** | **Factor label** |
| Sensation seeking | .92 |  |  |  |  |  |  | Self-control |
| Impulsivity/disinhibition | .81 |  |  |  |  |  |  |  |
| ADHD | * |  |  |  |  |  |  |  |
| Regular tobacco use |  | 1.00 |  |  |  |  |  | Tobacco (ab)use |
| Average tobacco using days |  | .43 |  |  |  |  |  |  |
| Ever used tobacco |  | .33 |  |  |  |  |  |  |
| Average alcohol units per month |  |  | .79 |  |  |  |  | Alcohol (ab)use |
| Regular alcohol use |  |  | .60 |  |  |  |  |  |
| Problematic alcohol use (AUDIT) |  |  | .55 |  |  |  |  |  |
| Average cigarettes per day |  |  |  | .76 |  |  |  | Tobacco (ab)use |
| Nicotine dependence (FTND) |  |  |  | .74 |  |  |  |  |
| Age alcohol initiation |  |  |  |  | .60 |  |  | Age at initiation |
| Age tobacco initiation |  |  |  |  | .43 |  |  |  |
| Age at first sexual intercourse |  |  |  |  | * |  |  |  |
| Ever other substance(s) |  |  |  |  |  | .70 |  | Lifetime substance use |
| Ever used cannabis |  |  |  |  |  | .56 |  |  |
| Age cannabis initiation |  |  |  |  |  |  | -.57 | - |
| Regular cannabis use |  |  |  |  |  |  | * |  |
| Age other substance initiation |  |  |  |  |  |  | * |  |

* Variable loading < .30

**D)** UKB data.

| **Factor** | **Eigenvalue** | **% of Variance** | **Cumulative %** |
| --- | --- | --- | --- |
| 1 | 2.35 | 16.80 | 16.80 |
| 2 | 1.53 | 10.93 | 27.72 |
| 3 | 1.47 | 10.47 | 38.20 |
| 4 | 1.18 | 8.42 | 46.61 |
| 5 | 1.04 | 7.46 | 54.07 |

| **Variable** | **Factor** | | | | |  |
| --- | --- | --- | --- | --- | --- | --- |
|  | **1** | **2** | **3** | **4** | **5** | **Factor label** |
| Regular tobacco use | .96 |  |  |  |  | Tobacco (ab)use |
| Ever used tobacco | .91 |  |  |  |  |  |
| Problematic alcohol use (AUDIT) |  | .77 |  |  |  | Alcohol (ab)use |
| Regular alcohol use |  | .71 |  |  |  |  |
| Average alcohol units per month |  | * |  |  |  |  |
| Nicotine dependence (FTND) |  |  | .70 |  |  | Tobacco (ab)use |
| Average cigarettes per day |  |  | .56 |  |  |  |
| Age tobacco initiation |  |  | * |  |  |  |
| Number of sexual partners |  |  |  | .57 |  | (Sexual) risk behavior |
| Age at first sexual intercourse |  |  |  | -.34 |  |  |
| Risk-taking proneness |  |  |  | * |  |  |
| Any behavioral/substance addiction |  |  |  |  | .52 | - |
| Ever used cannabis |  |  |  |  | * |  |
| Regular cannabis use |  |  |  |  | * |  |

* Variable loading < .30

**SUPPLEMENTARY TABLE S4**  The number of SNPs that was present in at least one sample in the analysis (#SNPs), the number of independent SNPs (with *R^2^*=0.1, LD merging distance 250kb, and a two-sided *p*<.1), and the name of the 10 independent SNP(s) with the smallest *p*-values and the corresponding bp position. SNPs that occur among the top-ten for multiple phenotypes are denoted with a superscript number. SNPs with a red superscript appear more than two times in primary phenotypes, green appear twice in primary phenotypes, and blue appear multiple times, but only once in a primary phenotype (the other time(s) in a factor)

| **Variable** | ***#SNPs*** | ***#independent SNPs*** | ***SNPs*** | ***bp*** |
| --- | --- | --- | --- | --- |
| **LIFETIME SUBSTANCE USE** | | | | |
| Ever used tobacco | 1,756 | 63 | rs1433708  rs11127908  rs12714592 ^1^  rs1248857 ^2^  rs1448602  rs1485582  rs113364248 ^3^  rs13319945  rs1248809  rs12637371 | 85481979  85869285  84387950  85018612  85780454  84688653  85741923  84025580  84934915  84219156  85472033  85893048  85018612  84808756  84424761  85487124  85468913  85135013  85143307  84048375  85086455  85841743  85409260  84668629  84210507  85153235  84299389  85381838  85465561  85345197 |
| Ever used cannabis | 6,383 | 83 | rs7636243  rs4473564 ^4^  rs1248857 ^2^  rs9868293  rs9856889  rs9821126  rs6804996  rs9832634  rs114226996 ^5^  rs9869787 |  |
| Ever used other substance(s) | 6,214 | 58 | rs74928832  rs79337314  rs74569441  rs60859036  rs116240880  rs4635723  rs9846520 ^6^  rs60407397  rs73136796  rs34584686 |  |
| **AGE AT INITIATION OF SUBSTANCE USE** | | | | |
| Age alcohol initiation | 6,215 | 41 | rs60538752  rs78355395  rs9816329  rs74332784  rs57547677  rs111993139  rs9861858  rs3887138  rs17501983  rs2123163 | 84043066  84906860  86115541  84313596  84981296  85266708  83998003  85648957  84833794  85243797 |
| Age tobacco initiation | 6,383 | 35 | rs10514735  rs12489914  rs7625608  rs9826386  rs79077228  rs7625199  rs13076735  rs55829275  rs74337284 ^7^  rs76446023  rs76578522  rs114612207  rs114382596  rs114226996 ^5^  rs980333  rs7622685  rs77687507  rs78495499  rs115454880  rs116593166 | 85798950  84987790  84964399  84265870  84093466  85721301  84160147  85804632  84564209  85997149 |
| Age cannabis initiation | 6,215 | 38 |  | 85392436  86063849  85683952  85143307  84838485  84692835  84171360  84586445  85628672  84356123 |
| Age other substance initiation | 6,215 | 37 | rs114228638  rs79526794  rs11713922  rs73136106  rs79664787  rs11127830  rs73147245  rs74355494  rs4261889  rs116230250 | 84028252  85006615  84294532  86070879  84498183  84024847  85759865  85258972  84741624  84991998 |
| **AVERAGE SUBSTANCE USE** | | | | |
| Average alcohol units | 6,383 | 69 | rs9839708 ^8^  rs9990096 ^9^  rs76363701  rs7611991 ^10^  rs7616936  rs80134033  rs78223691  rs111594685 ^11^  rs76517098  rs115217146  rs116772105 ^12^  rs7652808  rs9814165  rs73125330  rs9836564  rs55765801  rs115888131  rs77025486  rs62250660  rs17735321 ^13^ | 85058885  85411193  85436059  85759558  85803785  85816461  84976059  86070777  84303077  84906889 |
| Average cigarettes per day | 6,383 | 53 |  | 84202337  85603643  84223116  84288773  83997097  85685077  84498841  85830581  85446954  85113612 |
| Average tobacco using days | 6,215 | 25 | rs77114692  rs28366554  rs76813943  rs73141532  rs9876301  rs73130743 ^14^  rs12633811  rs111468253  rs12637767  rs114807292 | 85076445  84185799  83961148  85652614  85991634  85288268  83962883  84297075  85206681  85599240 |
| **REGULAR SUBSTANCE (AB)USE** | | | | |
| Regular alcohol use | 6,281 | 52 | rs12495758 ^15^  rs4301023  rs116559208 ^16^  rs114242255  rs79187939  rs13085678  rs75504236  rs375750  rs115730277  rs9872971 | 85554262  85057281  84462556  84201642  84191354  85358591  84030818  84987517  84173238  85344951 |
| Problematic alcohol use (AUDIT) | 6,379 | 67 | rs12495758 ^15^  rs1248860  rs4473564 ^4^  rs114375956  rs76873181  rs75902824  rs72919209  rs13060392  rs2326267  rs117898875  rs7650284  rs62261746 ^17^  rs7611991 ^10^  rs9834688  rs12714592 ^1^  rs515207  rs9835484  rs113364248 ^3^  rs6773147  rs9864886 | 85554262  85015779  85893048  85193798  84208185  84343551  85318295  84880419  85394420  84272987 |
| Regular tobacco use | 6,380 | 90 |  | 85472227  85958954  85759558  85035279  84387950  84926866  84742570  85741923  83988022  84133542 |
| Nicotine dependence (FTND) | 6,379 | 59 | rs73131909 ^18^  rs116383974  rs9990096 ^9^  rs114011108  rs116772105 ^12^  rs116240935  rs74745315 ^19^  rs79749903  rs9832119  rs78780329 ^20^ | 83960956  84576787  85411193  85693334  84202337  85285448  85012608  85249455  84972676  85100973 |
| Regular cannabis use | 5,676 | 50 | rs2044723  rs113817396  rs74337284 ^7^  rs114185016  rs1988552  rs2172846  rs2875889  rs1454089  rs4513464  rs6549016  rs1003985 ^21^  rs10049108 ^22^  rs10084664 ^23^  rs1013839 ^24^  rs1014796 ^25^  rs10154865 ^26^  rs1017638 ^27^  rs10212311 ^28^  rs10212377 ^29^  rs10212504 ^30^ | 85646002  83973343  84564209  84313475  85849851  85004637  85394742  84852101  83966741  85180270 |
| Any behavioral/substance addiction | 4,557 | 37 |  | 85870303  85702021  85964737  85243777  85544470  85409299  84751572  84297635  84299345  84299387 |
| **SEXUAL RISK BEHAVIOR** | | | | |
| Number of sexual partners | 5,677 | 54 | rs9824301  rs4856269  rs66680800  rs73132094 ^31^  rs114398534  rs62250579  rs62250575  rs73130743 ^14^  rs7646381  rs17735321 ^13^ | 85682888  85406735  85985324  84893889  84219421  85024652  84987256  85288268  85705615  85113612 |
| Sexual risk behavior ^a^ | NA | NA | NA  rs2044725  rs12714592 ^1^  rs62261746 ^17^  rs12714603  rs13316157  rs74843558  rs9839708 *^8^*  rs79314616  rs62255523  rs67028245 | NA  85645873  84387950  85958954  84656669  84115210  85655439  85058885  84801526  84804511  85394772 |
| Age at first sexual intercourse | 6,379 | 91 |  |  |
| **SELF-CONTROL** | | | | |
| Disinhibition | 6,211 | 45 | rs10212294  rs9816652  rs79361329  rs76062229  rs76508707  rs79874755  rs17437928  rs13088475  rs74446005  rs114307462 | 85668496  85067018  84594046  84211693  85397049  84116343  85406521  85019056  85733229  84764593 |
| Sensation seeking | 6,109 | 35 | rs1003984 ^32^  rs1003985 ^21^  rs1003986 ^33^  rs10049108 ^22^  rs10049397 ^34^  rs1005690 ^35^  rs10084664 ^23^  rs10084716 ^36^  rs1013839 ^24^  rs1014796 ^25^ | 85870199  85870303  85870363  85702021  84857250  85358806  85964737  85964639  85243777  85544470 |
| Risk-taking proneness | 4,557 | 69 | rs1003985 ^21^  rs10049108 ^22^  rs10084664 ^23^  rs1013839 ^24^  rs1014796 ^25^  rs10154865 ^26^  rs1017638 ^27^  rs10212311 ^28^  rs10212377 ^29^  rs10212504 ^30^ | 85870303  85702021  85964737  85243777  85544470  85409299  84751572  84297635  84299345  84299387 |
| ADHD | 3,436 |  | rs17023190  rs111594685 ^11^  rs114459574  rs73132094 ^31^  rs7615964  rs116559208 ^16^  rs77423946  rs3860559  rs112117757  rs17021771 | 85752400  86070777  84931784  84893889  85660567  84462556  85644855  84765953  84525528  84796650 |
| **FACTORS** | | | | |
| Lifetime substance use (meta-analysis) | 2,464 | 12 | rs6419760  rs60750563  rs116351045  rs77430012  rs2171140  rs75892230  rs6764254  rs61555026  rs73843277  rs9864651 | 84870987  84489985  85507460  85473111  85126955  85506121  84613365  84685842  85122712  85514116 |
| Tobacco (ab)use (meta-analysis) | 6,352 | 44 | rs73131909 ^18^  rs9863620  rs13059122  rs9846520 ^6^  rs78780329 ^20^  rs12629798  rs62250718  rs1874866  rs17735321^13^  rs74745315 ^19^ | 83960956  85448493  84181443  84299389  85100973  85905591  85523783  84988633  85113612  85012608 |
| Lifetime smoking (UKB) | 4,557 | 81 | rs1003985 ^21^  rs10049108 ^22^  rs10084664 ^23^  rs1013839 ^24^  rs1014796 ^25^  rs10154865 ^26^  rs1017638 ^27^  rs10212311 ^28^  rs10212377 ^29^  rs10212504 ^30^ | 85870303  85702021  85964737  85243777  85544470  85409299  84751572  84297635  84299345  84299387 |
| Regular alcohol use (UKB) | 4,557 | 64 | rs1003985 ^21^  rs10049108 ^22^  rs10084664 ^23^  rs1013839 ^24^  rs1014796 ^25^  rs10154865 ^26^  rs1017638 ^27^  rs10212311 ^28^  rs10212377 ^29^  rs10212504 ^30^ | 85870303  85702021  85964737  85243777  85544470  85409299  84751572  84297635  84299345  84299387 |
| Self-control (NTR) | 6,109 | 45 | rs1003984 ^32^  rs1003985 ^21^  rs1003986 ^33^  rs10049108 ^22^  rs10049397 ^34^  rs1005690 ^35^  rs10084664 ^23^  rs10084716 ^36^  rs1013839 ^24^  rs1014796 ^25^ | 85870199  85870303  85870363  85702021  84857250  85358806  85964737  85964639  85243777  85544470 |

^a^ Not available in the SNP-based meta-analysis

**SUPPLEMENTARY FIGURE S1** **(A)** Power as a function of sample size (*N*) and effect size (%*R^2^*) **(B)** as well as reported in more detail for sample sizes below 10,000.

**A)**
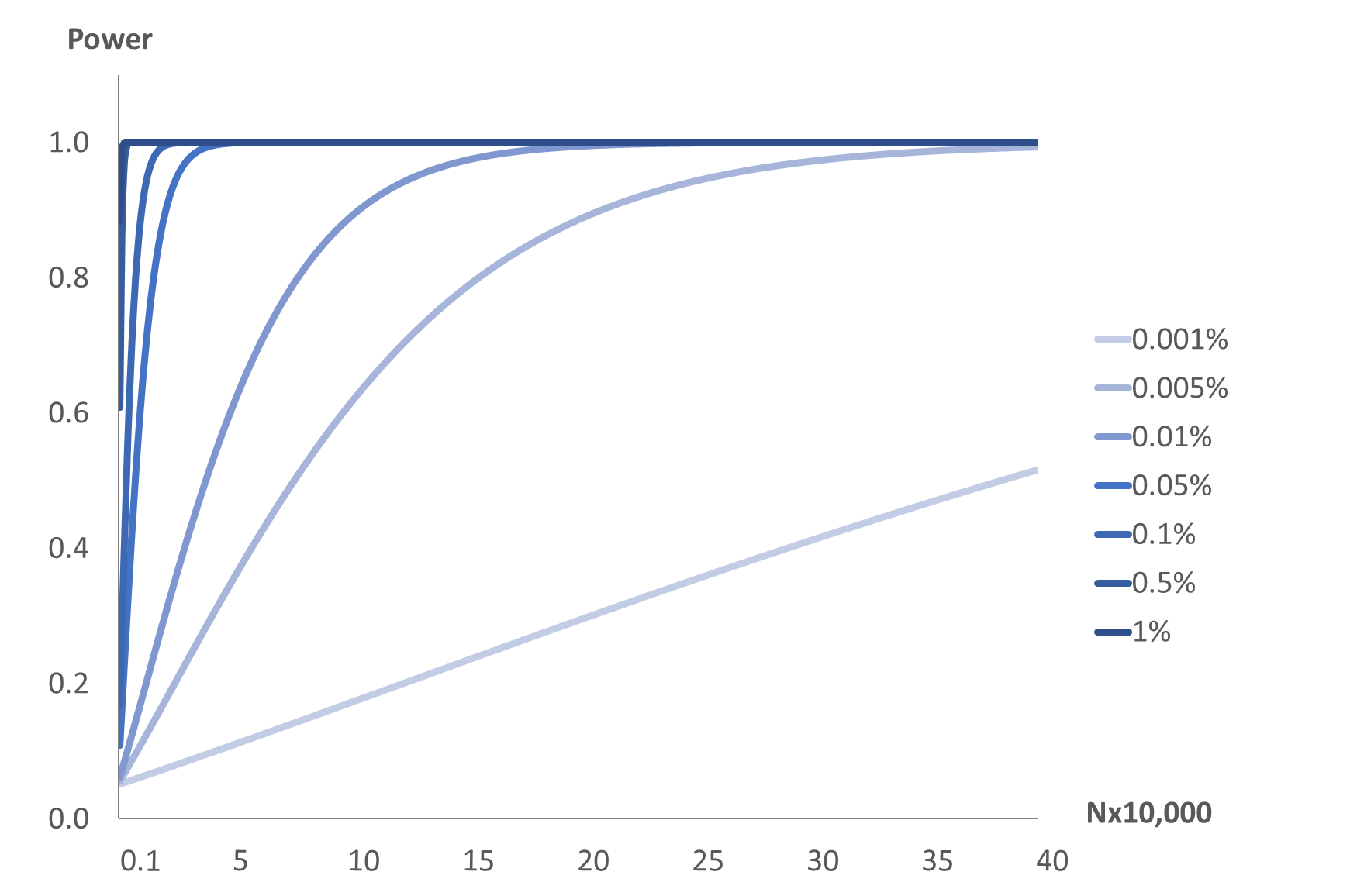


**B)**


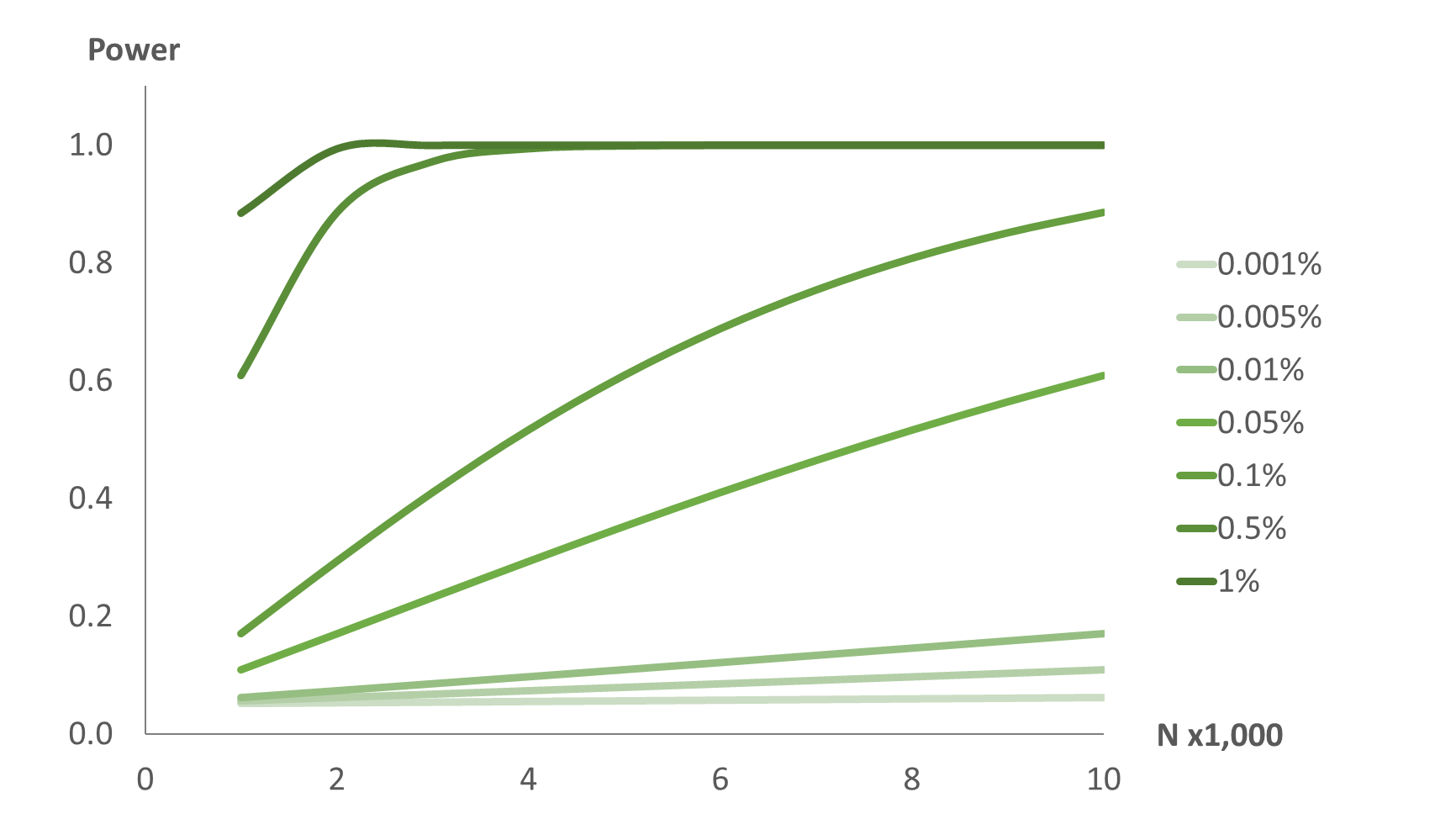


**SUPPLEMENTARY FIGURE S2** Frequency distribution of the basepair positions of the independent SNPs that were most strongly associated with the included risk behavior phenotypes with **(panel A)** showing the distribution in 10 equal-sized bins (330,000 bp), **(panel B)** showing the distribution in 3 bins (780,000). Most of the associated top SNPs fell in the bins between 84,950,956 and 85,940,956, which is a region containing many cis eQTLs **(panel C)**

**Panel A)**


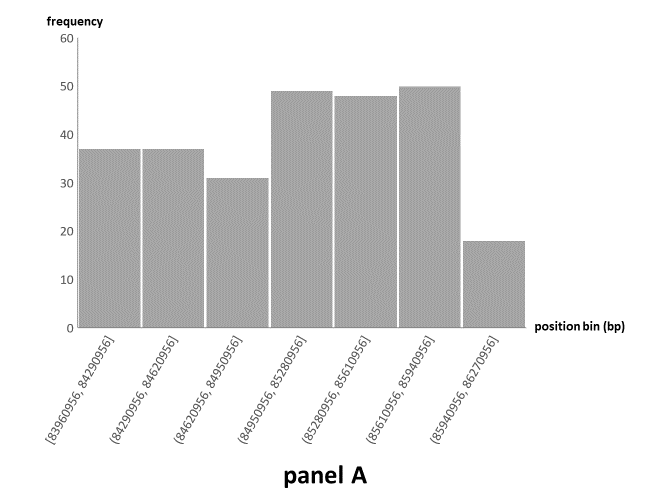


**Panel B)**


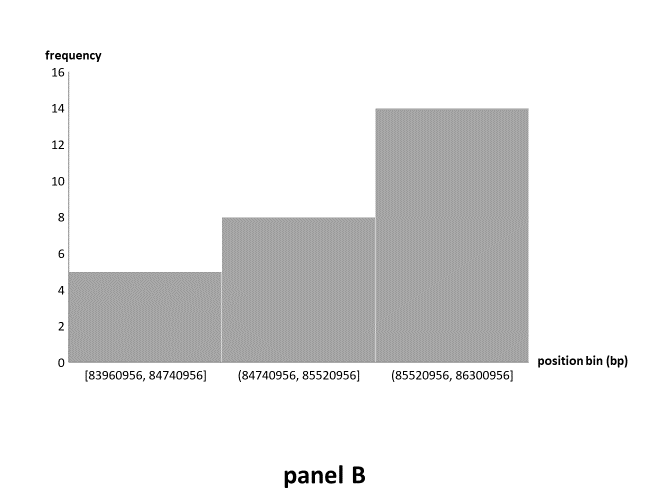


**Panel C)**


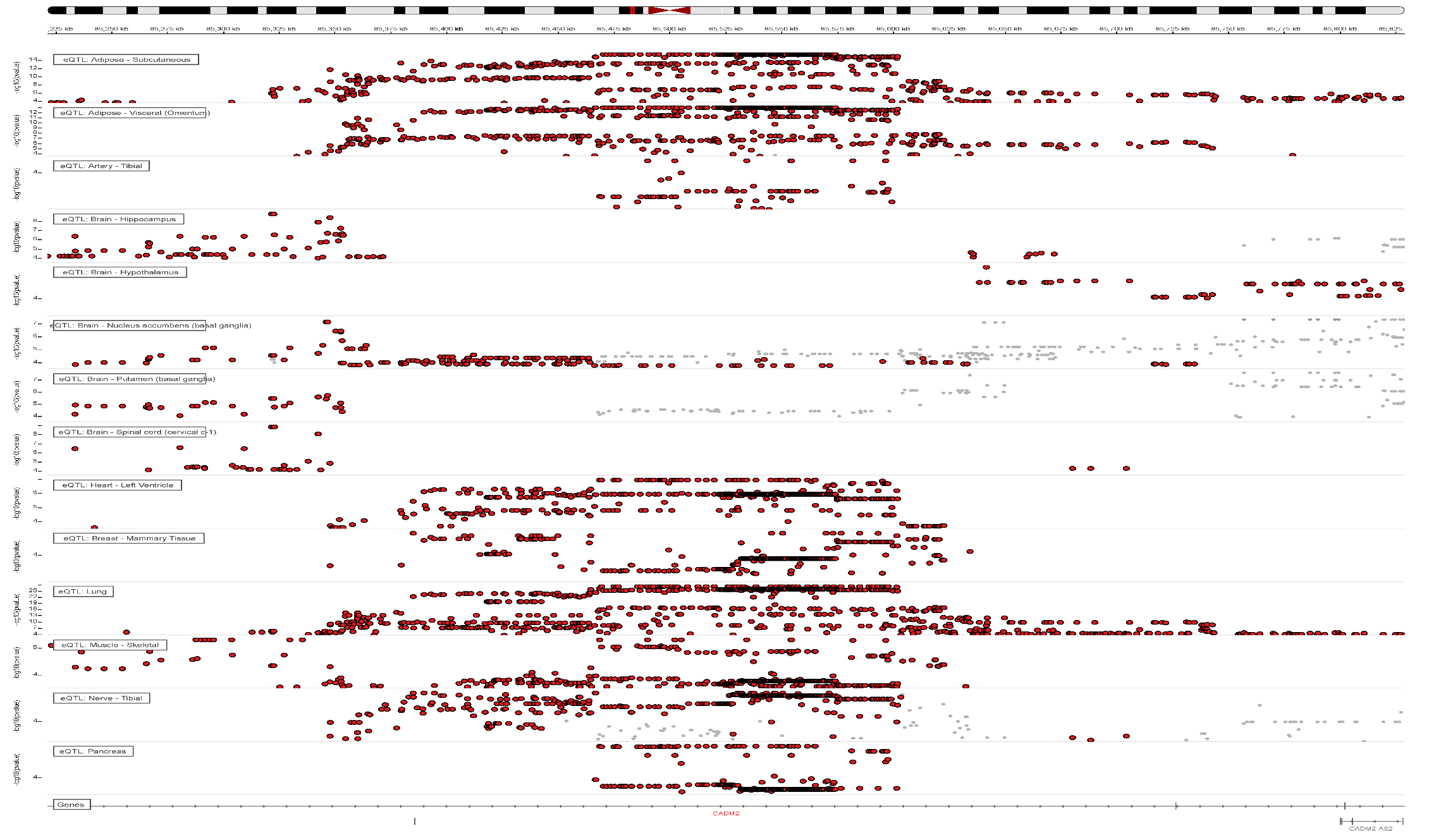


**LITERATURE**

1. Mitchell BL, Campos AI, Rentería ME, et al. Twenty-Five and Up (25Up) Study: A New Wave of the Brisbane Longitudinal Twin Study. *Twin Research and Human Genetics* 2019:1-10.

2. Dick D, Nasim A, Edwards AC, et al. Spit for Science: launching a longitudinal study of genetic and environmental influences on substance use and emotional health at a large US university. *Frontiers in genetics* 2014;5:47.

3. Willemsen G, Vink JM, Abdellaoui A, et al. The Adult Netherlands Twin Register: twenty-five years of survey and biological data collection. *Twin Research and Human Genetics* 2013;16:271-281.

4. Sudlow C, Gallacher J, Allen N, et al. UK biobank: an open access resource for identifying the causes of a wide range of complex diseases of middle and old age. *PLoS Med* 2015;12:e1001779.

5. Pasman JA, Verweij KJ, Gerring Z, et al. GWAS of lifetime cannabis use reveals new risk loci, genetic overlap with psychiatric traits, and a causal effect of schizophrenia liability. *Nature neuroscience* 2018;21:1161-1170.

6. The 1000 Genomes Project Consortium. A global reference for human genetic variation. *Nature* 2015;526:68-74.

7. Shim H, Chasman DI, Smith JD, et al. A multivariate genome-wide association analysis of 10 LDL subfractions, and their response to statin treatment, in 1868 Caucasians. *PLoS One* 2015;10:e0120758.

8. Dudbridge FJPg. Power and predictive accuracy of polygenic risk scores. 2013;9.

9. Gillespie NA, Henders AK, Davenport TA, et al. The Brisbane Longitudinal Twin Study: pathways to Cannabis Use, Abuse, and Dependence project—current status, preliminary results, and future directions. *Twin Research and Human Genetics* 2013;16:21-33.

10. Peterson RE, Edwards AC, Bacanu SA, Dick DM, Kendler KS, Webb BT. The utility of empirically assigning ancestry groups in cross‐population genetic studies of addiction. *The American journal on addictions* 2017;26:494-501.

11. Bycroft C, Freeman C, Petkova D, et al. The UK Biobank resource with deep phenotyping and genomic data. *Nature* 2018;562:203.

12. Kessler RC, Adler L, Ames M, et al. The World Health Organization Adult ADHD Self-Report Scale (ASRS): a short screening scale for use in the general population. *Psychol Med* 2005;35:245-256.

13. Saunders JB, Aasland OG, Babor TF, De la Fuente JR, Grant M. Development of the alcohol use disorders identification test (AUDIT): WHO collaborative project on early detection of persons with harmful alcohol consumption‐II. *Addiction* 1993;88:791-804.

14. Heatherton TF, Kozlowski LT, Frecker RC, FAGERSTROM KO. The Fagerström test for nicotine dependence: a revision of the Fagerstrom Tolerance Questionnaire. *Br J Addict* 1991;86:1119-1127.

15. Lynam DR, Smith GT, Whiteside SP, Cyders MA. The UPPS-P: Assessing five personality pathways to impulsive behavior. *West Lafayette, IN: Purdue University* 2006.

16. Zuckerman M. The sensation seeking scale V (SSS-V): Still reliable and valid. *Personality and Individual Differences* 2007;43:1303-1305.

17. Tinsley HE. Uses of factor analysis in counseling psychology research. 1987;34:414.
